# Sport for a Livelihood and Well-being: From Leisure Activity to Occupational Devotion

**DOI:** 10.1007/s41978-021-00091-6

**Published:** 2021-07-31

**Authors:** Kirstin Hallmann, Laura Bogner, Kathrin Sander, Konrad Reuß

**Affiliations:** Institute of Sport Economics and Sport Management, German Sport University Cologne, Am Sportpark Muengersdorf 6, 50933 Cologne, Germany

**Keywords:** Lifestyle entrepreneurs, Occupational devotion, Well-being, Quality of life, Sports industry

## Abstract

This study explored the occupational devotion of lifestyle entrepreneurs and their well-being. Although the number of enterprises in the sports industry increased over the past years, limited literature exists on this topic. Therefore, this paper focused on lifestyle entrepreneurs who turned their sports into their occupations. We conducted semi-structured interviews and a follow-up survey with lifestyle entrepreneurs (*N* = 13) from various sports (e.g., yoga, kiting, football). The thematic analysis revealed a strong connection between the individuals’ choice of career and well-being. Other themes (and sub-themes), such as mental and physical health and value (co-)creation, were identified, corroborating the authors’ assumption that these lifestyle entrepreneurs started a career in their sports to reach a higher quality of life. The results uncovered that well-being and occupational devotion are closely linked. Co-creation is connected to well-being, and this can accrue social capital in the community. Thus, the results recommended support for lifestyle entrepreneurs as they provide community benefits.

## Introduction

“I can say that yoga always equals happiness to me – every lesson that I teach. I really enjoy being a yoga teacher. I like practicing it, too, but being a yoga teacher is so much better” (Peter, yoga). This quote highlights the extent to which lifestyle entrepreneurs value their occupation within the sports industry. Simultaneously, the quotation shows appreciation for sports as a leisure activity. Peter is a lifestyle entrepreneur. Ski instructors, owners of surf schools, or personal coaches are all lifestyle entrepreneurs. Lifestyle entrepreneurs are “individuals who own and operate businesses closely aligned with their personal values, beliefs, interests, and passions” (Marcketti et al., 2006, p. 241). They can also work as a freelancer (Deakins & Freel, 2006).

Entrepreneurship is a dynamic concept (Howorth et al., 2005) and an instrument for personal and organizational development (Ball, 2005). Ratten (2010) highlighted that there is no standard definition of entrepreneurship or the entrepreneur. Entrepreneurs can be best defined by what they do (Gartner, 1990; Howorth et al., 2005; Ratten, 2010). They innovate, take risks, control, are committed, have a vision, and are proactive (Gartner, 1990; Howorth et al., 2005; Ratten, 2010; Schumpeter, 1934). Entrepreneurs define themselves via their own organization (Howorth et al., 2005). This also applies to entrepreneurs in the sports business.

Lifestyle entrepreneurs pursue their work to balance their social, economic, and value aspirations (Ateljevic & Doorne, 2000). They are also highly devoted to their sports activity and pursue it seriously. They demonstrate skills and experience, creativity, and aptitude while being in control and finding themselves in a specific social milieu (Stebbins, 2004a, 2009) linked to their sports.

Ratten (2020a) described the aspiration for happiness and a good work-life balance as the primary diver of becoming a lifestyle entrepreneur. Lifestyle entrepreneurs focused on their quality of life by following their passion as a business activity rather than focusing on the financial outcome (Ratten, 2020a). Consequently, boundaries between leisure and work are distorted for lifestyle entrepreneurs (Andersson Cederholm, 2015) as their leisure activity transforms into an occupation.

Entrepreneurship within specific business sectors gained growing research interest, for example, sport entrepreneurship (Ratten, 2010, 2011, 2020a, b). The number of sports firms, particularly those of lifestyle entrepreneurs, grew (Jones et al., 2017). Self-employment became a frequent option for many “due to lifestyle reasons and their affinity with sport” (Jones et al., 2017, p. 227). Wright and Wiersma (2020) outlined associations between occupational devotion and lifestyle entrepreneurship. Studies explored lifestyle entrepreneurs from an identity or economic motivational perspective (Peters et al., 2009). Yet, other areas such as lifestyle entrepreneurs’ occupational devotion or personal well-being remained unexplored.

Thus, Jones et al. (2017) called for further research regarding individual motivation to pursue a profession within the sports business and their (dynamic) attachment towards their occupational devotion. This study also resonates with Ratten and Tajeddini’s (2019) call for more research about the extent of sport entrepreneurship and its diverse forms of economic, social, and cultural capital. Therefore, this study explores and provides insight into the serious leisure consumption of individuals classified as lifestyle entrepreneurs within the sports business. They choose to transform their leisure activities into an occupation for a better work-life balance (Ratten, 2018; Wallis et al., 2020). More people strive to improve their employment situation to increase their quality of life and, thus, their well-being (Ateljevic & Doorne, 2000; Wang et al., 2019).

Consequently, this study will tap into this gap of not knowing how this choice of profession contributes to lifestyle entrepreneurs’ well-being. Furthermore, the decision to become a lifestyle entrepreneur, looking at elements of quality of life such as happiness and health, will be explored. Thereby, we look into the transition from sports as a serious leisure activity to occupational devotion. Therefore, we investigated the following research questions: *RQ1:* What are the motivations to become a lifestyle entrepreneur and exchange the leisure activity for a professional career? *RQ2*: To what extent does the career as a lifestyle entrepreneur contribute to individual well-being? *RQ3*: What is the lifestyle entrepreneur’s work-life balance?

The results will help support agencies of entrepreneurs at the community level and policymakers to better understand lifestyle entrepreneurs in the sports business (and in other leisure areas, such as culture). For instance, agencies specialized in self-employment, employment agencies, non-profit organizations for founders, or consulting firms can provide more customized services and consultations for them. Moreover, sport entrepreneurs are innovative so that lifestyle entrepreneurs become competitive drivers in the sports sector (Ratten & Ferreira, 2017; Ratten, 2020b), which can trickle down, for instance, to the non-profit sector. Sport entrepreneurship is essential during times of crises (Ratten, 2020b) such as COVID-19. Sport entrepreneurs (and thus lifestyle entrepreneurs) can show larger organizations how to cope with crises (Ratten, 2020b), notwithstanding that sport entrepreneurs are very vulnerable themselves.

## Theoretical Framework and Literature Review

### Lifestyle Entrepreneurship

There were various interpretations of so-called lifestyle entrepreneurs, as this concept was complex (Marcketti et al., 2006; Wallis et al., 2020). Henderson (2002) stated that lifestyle preferences rather than economic benefits usually drive lifestyle entrepreneurs. They wanted to make a decent living but under certain self-determined conditions, such as seeking “independence and control over their own schedule” (Henderson, 2002, p. 49) or reaching personal goals (Lewis, 2008). Values and interests drove lifestyle entrepreneurs to run their business accordingly (Henricks, 2002; Marcketti et al., 2006).

Another key feature was a general indifference towards growth among lifestyle entrepreneurs. Whereas traditional entrepreneurs generally focused on growth and economic success (Marchant & Mottiar, 2011), lifestyle entrepreneurs did not actively seek these features (Wallis et al., 2020). Lifestyle entrepreneurs not even showed entrepreneurial intentions (Wallis et al., 2020) but defined their professional success in relation to family, community services, and quality of life (Marcketti et al., 2006). Their main drive derived from making a decent living, allowing them to enjoy a certain quality of life while earning (just) enough money to survive (Deakins & Freel, 2006; Morrison et al., 1999). Through the constant development of entrepreneurship, lifestyle entrepreneurship also included non-profit activities (Ratten, 2020a).

Various scientists also added another personal dimension to their definition of a lifestyle entrepreneur (Andersson Cederholm, 2015; Marcketti et al., 2006) by explaining that they made their hobby their business (Wallis et al., 2020). By doing so, they simultaneously wished to find a better balance between their personal and professional life (Ratten, 2018; Wallis et al., 2020). Independence, free will, autonomy, control, flexibility, and individuality represented additional motivational factors (Ratten, 2018). Thus, lifestyle entrepreneurs pursued their chosen lifestyle as part of their work environment by creating their own companies (Ratten, 2018, 2020a).

Jones et al. (2017) noted that a growing number of people in the sports industry were embracing self-employment. They attributed this change to the fact that lifestyle choices gained importance among employment criteria. The factor “quality of life” played a significant role for this type of entrepreneur (Marcketti et al., 2006; Peters et al., 2009). Lifestyle entrepreneurs considered pursuing their passion while earning their income as a quality of life (Ratten, 2020a). Passion was a driver of entrepreneurship (Guercini & Ceccarelli, 2020). However, quality of life was a subjective experience based on individual expectations and beliefs (Peters et al., 2009). It involved financial aspects just as much as emotional or somatic dimensions.

Although in recent years, several authors studied lifestyle entrepreneurs in the tourism sector (Ateljevic & Doorne, 2000; Bredvold & Skålén, 2016; Fadda, 2020; Marchant & Mottiar, 2011; Peters et al., 2009; Ratten, 2018; Walmsley, 2019). Only a few exceptions explored the phenomenon of lifestyle entrepreneurship concerning sport (González-Serrano et al., 2020; Jones et al., 2017, 2020; Ratten, 2010, 2011; Wallis et al., 2020) or other sectors (Andersson Cederholm, 2015; Marcketti et al., 2006). Wallis et al. (2020) even claimed that it is not yet fully understood.

### The Serious Leisure Perspective and Occupational Devotion

Stebbins introduced serious leisure in the early 1970s. It continued to be relevant for academic research (Stebbins, 2007, 2009). This theoretical framework divided leisure into three categories: serious, project-based, and casual leisure. Leisure was described as an uncoerced activity during free time, resulting in satisfaction or fulfillment for individuals (Stebbins, 2009). Of the three leisure categories, serious leisure received the most attention from academia. Stebbins (1992) provided the following definition: It is “the systematic pursuit of an amateur, hobbyist, or volunteer activity that is sufficiently substantial and interesting for the participant to find a career there in acquisition of its special skills and knowledge” (p. 3).

Six qualities applied to participants in serious leisure activities: 1) the need to persevere; 2) finding a leisure career in the endeavor; 3) significant personal efforts, such as training or experience; 4) durable benefits, such as self-actualization or feeling of belongingness; 5) unique ethos and social world; and 6) strong identification with the chosen pursuits (Stebbins, 2007, 2009).

As part of the serious leisure perspective, Stebbins’ concept of occupational devotion further explored the relationship between work and leisure (Stebbins, 2004a, 2009). He claimed that occupational devotees barely draw a line between these two aspects of their lives while experiencing their work with positivity (Stebbins, 2004a). Consequently, occupational devotees distinguished free time in which leisure occurs typically from work. Therefore, occupational devotion was defined by “a strong, positive attachment to a form of self-enhancing work, where the sense of achievement is high, and the core activity (set of tasks) is endowed with such intense appeal” (Stebbins, 2004a, p. 2). This devotion applied to the individual’s work and affected their lifestyle, actions, motivation, and relationships (Stebbins, 2004a, 2009), including their identity and how others see them (Wright, 2020). To further explain the concept of occupational devotion, Stebbins (2004a) listed six criteria fitting the theoretical framework of the serious leisure perspective. These features included skill, knowledge or experience; variety; creativity or innovativeness; control; aptitude and taste; physical and social milieu (Stebbins, 2004a).

Occupational devotees were driven by an intrinsic appeal towards their personal and professional goals while valuing their freedom to make their own decisions (Stebbins, 2004a) and turning their devotion into “a part of their personal and social identity” (Wright & Wiersma, 2020, p. 26). According to Neulinger (1981), leisure was one channel to express and establish a person’s desire for liberty, fulfillment, and self-actualization. These serious leisure components, along with other durable benefits or personal development, pointed towards one significant factor essential to the concept of occupational devotion: quality of life (Stebbins, 2004b).

### Occupational Devotion and Lifestyle Entrepreneurship in the Sports Sector

Both occupational devotion and lifestyle entrepreneurship show various similarities. They emphasized personal motives, such as lifestyle, values, or fulfillment, over economic factors for choosing one’s career path (Stebbins, 2009; Wallis et al., 2020). The boundaries between work, personal life, and leisure become increasingly blurred in both concepts (Andersson Cederholm, 2015; Wallis et al., 2020). The lack of boundaries could be even more emphasized in the sports industry as it consisted of an emotional dimension and differs from other sectors (González-Serrano et al., 2020; Jones et al., 2017). The emotional dimension related to the opportunity of value co-creation as production and consumption processes take place simultaneously.

Value creation and value co-creation were two common themes in academic research on entrepreneurship over the past decades (Chiu et al., 2019; Grönroos et al., 2015; Kelleher et al., 2019). Entrepreneurs created value by offering their products and services while interacting with multiple stakeholders (Grönroos et al., 2015; Kelleher et al., 2019). Thus, value was co-created. Customers were at the core of this process (Chiu et al., 2019; Uhrich, 2014). In her study on sport-based entrepreneurship, Ratten (2010) closely examined sports and value creation dynamics. She argued that value creation is essential for entrepreneurs in the sports industry and suggested links to social responsibility (Ratten, 2010). Sports contributed to social responsibility by supporting peace, political stability, and health (Smith & Westerbeek, 2007). Sports-based enterprises emphasized social capital over economic objectives making them more competitive (Ratten, 2010). Sports and entrepreneurship were innovative and constantly adapted to change, and consequently, lifestyle entrepreneurs became competitive drivers in the sports sector (Ratten, 2020b).

Whereas Stebbins (2004a) stated that occupational devotion is relatively uncommon, newer trends regarding ideas, structures, and work environment decisions gained popularity. A growing number of people negotiated the terms of their current work situation to improve their quality of life in terms of well-being and a healthy work-life balance (Ateljevic & Doorne, 2000; Wang et al., 2019). Well-being described an individual’s subjective perception of a pleasant life, including positive moods, pleasant emotions, and pleasure (Diener & Seligman, 2004). Subjective well-being related to all life components, such as societal conditions, income, physical health, social relationships, or work (Diener & Seligman, 2004). Satisfaction with life, the experience of pleasant emotions, the engagement in interesting activities, and the absence of negative emotions or pain characterized abundant subjective well-being (Diener, 2000). Engaging in activities that enable self-fulfillment also contributed to the perception of a good life (Huta & Waterman, 2014; Waterman, 2011).

Andersson Cederholm (2015) found that lifestyle entrepreneurs constantly negotiated boundaries between different life spheres (leisure and work). In traditional forms of employment these spheres differed but these spheres blurred for lifestyle entrepreneurs (Andersson Cederholm, 2015). These boundaries include everyday aspects, such as social and professional relationships, home, business, work, family, and leisure (Andersson Cederholm, 2015). Sun et al. (2020) even highlighted that lifestyle entrepreneuers considered the occupation not as work but as a lifestyle.

Even though most research recognized that lifestyle entrepreneurs were mainly motivated by personal and lifestyle reasons, Marchant and Mottiar (2011) claimed that their motives and goals could change: While initially, the lifestyle component was at the core of their business, the economic element shifted into focus over time due to changes in the internal (e.g., family commitments) or external environment (e.g., general economic growth; Marchant & Mottiar, 2011). Lifestyle entrepreneurs had a dual identity: a sports persona and a business persona. These identities interacted and were complementary (Wallis et al., 2020). The dual identity created trust among the customers and facilitated authenticity in their community (Wallis et al., 2020).

## Methods

### Instrumentation

This study evaluated the motivation of lifestyle entrepreneurs, their work-life balance, and personal well-being. A qualitative approach was the most suitable research paradigm as we considered individual views, preferences, and attitudes. We comducted semi-structured interviews. Mainly open-ended questions supported the acquisition of such personal insights. This study’s interview guideline was initially developed to explore overall subjective experiences in sports, including serious leisure and flow, in a larger research project. In this context, the interview guideline was tested, validated, and adjusted until data collection could start. The interview guideline, relating to this study’s purposes, was informed by the serious leisure framework (Stebbins, 1982). It allowed the examination of the participants’ relation to sports and how they experienced it. The interview started with general questions relating to their sports activity (*What does your sport mean to you? How regularly do your practice? How intensively do you practice?).* These questions were also generally related to the serious leisure framework.

Moreover, we included specific questions about the sports experiences (e.g., *Have you experienced particular moments with intense joy or happiness because of your sport? Can you describe a situation in which you have experienced this feeling*?). These questions indicated the respondents’ well-being and were related to their arousal. Further, we added questions relating to the respondents’ flow (e.g., *Could you describe the term flow?*) during sports participation and learnings (e.g., *Would you say that you have changed over time due to sports experiences?*). Follow-up questions, if deemed necessary, were also included to understand the individual’s perceptions of their sport.

Additionally, lifestyle entrepreneurs within the sports business received a short questionnaire. Thereby, we collected more profound insights into the participants’ occupational devotion (Stebbins, 2004a, 2009). Particularly, we examined the nature of their occupational devotion. The questionnaire included a short overview of their sports background (*At what age did you start with your sport? When did you start turning your leisure activity into a (form of) profession (main occupation or secondary occupation?*), motivation, and reasons for a career in the sports business (*What was your motivation for this shift?).* Like the interview guideline, the questionnaire consisted predominantly of open-ended questions and a list of the dimensions defining occupational devotion. The respondents indicated whether this dimension applied to them and their occupation or not. Moreover, they were to name the most important dimension and explain the reason for their choice. This should highlight the dimension’s relevance for the participants’ entrepreneurship.

### Data Collection and Sampling

The in-depth interviews took place between the 9^th^ of June and the 4^th^ of September 2020. They lasted between 12:53 and 24:11 min. We conducted all interviews via telephone or video call due to the restrictions of social contacts during the COVID-19 pandemic. The interviewwes replied to the additional questionnaire in written form (in October 2020). The spoken language in the interviews and the questionnaire was German. Informed consent included permissions of recording and data usage. The participants were initially selected through the authors’ extensive personal network. Further, snowball sampling was applied as a non-probability sampling approach to recruit participants. This study’s inclusion criteria included being engaged in serious leisure, pursuing an individual sport, a team sport,or a sport with an animal, and heterogeneity concerning age and gender.

### Participant Characteristics

The final sample for this study consisted of *N* = 13 lifestyle entrepreneurs. Eleven of the thirteen interviewees completed the questionnaire. Participants were between 23 and 60 years old. 61.5% female and 38.5% male participants displayed the gender structure of this sample. Most of them were German (*n* = 11), one was German-American, and one participant was Austrian. They covered a range of sports in which they are active, such as yoga, football, basketball, kitesurfing, running, tennis, or rescue dog work. The participants were classified within their entrepreneurship as coaches or instructors (*n* = 8) and professional athletes (*n* = 2). Others work in a broader sports context (*n* = 3) as managers of a climbing gym, sports content creators, or technical product managers. The participants’ characteristics, as presented in Table 1, cover various forms of entrepreneurship within the sports sector.

**Table 1:** Characteristics of the study sample

ID	Participant (synonym)	Age	Gender	Sport	Involvement in the sport started at age	Shift to occupational devotion started at age	Title
1	Peter	58	m	Yoga	-	-	Yoga teacher
2	Sarah	28	f	Football	12	24	Professional football player
3	Nicole	54	f	Yoga	-	-	Yoga teacher
4	Adam	46	m	Basketball	15	22	Basketball coach
5	Lina	29	f	Kiting	22	27	Technical product manager (former kite instructor)
6	Anne	42	m	Running	15	30	Sport Content Creator
7	Stephan	52	m	Climbing	12	24	Manager of a climbing gym
8	Benjamin	32	m	Running	20	26	Professional trail runner
9	Karen	60	f	Rescue dog work	40	50	Man trailing instructor
10	Leonie	23	f	Yoga	12	20	Yoga teacher
11	Julia	39	f	Kiting	27	30	Kite instructor and school manager
12	Robin	35	m	Tennis	6	18	Tennis lecturer & coach
13	Mona	36	f	Yoga	30	35	Yoga teacher

### Data Analysis

The integration of several theories related to the examination of experiences in sports primarily structured the interview guideline. Data analysis was, therefore, theory-driven and mainly based on thematic coding. The serious leisure framework (Stebbins, 1982) and occupational devotion (Stebbins, 2004a, 2009) informed this study part. These themes led to suitable codes for data analysis and sub-themes.

We developed further sub-themes inductively, that is, empirics driven, through iterative reading. The transcribed spoken data served as the basis for continuative analysis, whereas the additional questionnaire provided additional insights into themes and codes for a purposeful analysis. The combination of a theory- and empirics-driven approach led to a comprehensive category system. This system served as the basis for computer-supported data analysis as interview statements could easily be structured. The data were collected and transcribed in the German language. The analysis was executed in the German language, and selected quotes and statements were translated into English. Table 2 presents one exemplary part of this category system for the theme of well-being with corresponding interview excerpts.

**Table 2:** Overview about themes, sub-themes and sample quotations

Theme	Sub-Theme	Quote
Well-Being	Mental & Physical Health	“I realized that you can draw a lot of strength from this positive energy that you experience through your sport and its moments of joy for your everyday life and your work.” (Adam, basketball)
	Happiness	“It’s my fun factor. It is what I really enjoy doing with the dogs. I love working with my dogs.” (Karen, rescue dog work)
	Self-fulfillment	“To me, football is something that defines my everyday life, my moods, my thoughts, my emotions, like nothing else in my life. At the moment, this is my highlight. All of these emotions that you put into it, the effort you put into it. It’s more than an experience. It fulfills and defines me completely.” (Sarah, football)

### Trustworthiness

Like quantitative research, qualitative studies aim to present findings that result from high-quality research and are, therefore, regarded as trustworthy. Lincoln and Guba (1985) defined credibility, transferability, dependability, and confirmability as quality criteria. Within the research team, peer debriefing took place regularly. Therefore, all research steps, including the development of the interview guide and codebook, were discussed and validated. A prolonged engagement with the initial broader research aim in combination with this study’s focus supported credibility.

Furthermore, the whole research process was documented transparently. This documentation, along with descriptions of the thematic focus of this study, the context of data collection, and the presentation of the given samples, helped to access the transferability of findings to other contexts. One member of the research team coded the entire material. However, there was a continuous exchange regarding the coding with all other members to ascertain dependability.

## Results and Discussion

All participants were intensively engaged in their sports. Following Stebbins (2007) categorization of leisure, sports consumption can be defined as serious leisure. The respondents’ participation exceeded project-based and casual leisure as their sports played a substantial role in their lives. It not only determined their leisure and work time but also shaped their social and personal identity (Stebbins, 2007, 2009). Julia stated that “It [kitesurfing] truly defined me”. Sarah explained that football determined her everyday life, her mood, her thoughts, and emotions.

Further, a strong identification with their leisure activity came to the fore. Statements such as “climbing is my life” (Stephan), “tennis is a huge part of my life” (Robin), or “I have lived only for the sport” (Julia, kitesurfing) supported the idea that sports participation can be understood as serious leisure. The interviewees mentioned often that their sports engagement and devotion shaped their personal and social identity (Wright, 2020) accompanied by cultural values and lifestyle aspects. This is linked to occupational devotion.

The participants’ unique ethos and social world were also influenced and formed through characteristics of their leisure activities. Every sports had its community or sub-culture (Stebbins, 2007, 2009). Stephan described this for climbing as “you are a big family and simply meet everywhere. Over and over again you meet the same people, and you like each other because they have the same passion as I have and that matters”. The interviewees mentioned the social environment and the exchange with others who shared the same interests as essential aspects determining how they experienced their sports.

Our participants put a lot of effort into their sports consumption. They all trained regularly and had competitions on weekends or did longer trips and projects. Through their significant personal effort, they constantly gained skills and knowledge, made new experiences, and delivered good performances. Lifestyle entrepreneurs strived to pursue higher skills (Guercini & Ceccarelli, 2020). They also faced difficulties or challenges, especially when accomplishing new projects (climbing), a marathon (running), new jumps (kitesurfing), or body positions (yoga). Participants across all observed sports mentioned a need to persevere. To succeed, participants spoke of pushing themselves over their boundaries (Nicole, yoga) or about repeating and not giving up until they make it (climbing, kitesurfing). Overcoming such challenges through perseverance left the participants with positive feelings. Effort and perseverance were strongly connected to themes of self-actualization and self-fulfillment and contributed to well-being.

All participants mentioned that they received various durable benefits (e.g., personal growth) from their serious leisure consumption. Climbing helped to gain self-confidence (Stephan), basketball enhanced positive energy (Adam). In contrast, yoga helped all participants calm down, relax, and develop physically within practice and beyond. Regarding health benefits, participants reported lower stress levels (Anne, running; Peter, Yoga). Most participants mentioned good feelings, body improvements, or aspects of self-actualization through their sports from which they could also benefit in other areas of their lives.

During their sports engagement, the participants found a leisure career in the endeavor, another quality of serious leisure. All participants found a profession in the sports industry, though their job conditions vary considerably. They worked full-time or part-time; were self-employed, employed by a company, or worked with no monetary compensation at all. This covered a broad range of entrepreneurship, including for-profit and non-profit activities (Ratten, 2020a). They covered various sports and occupations: Four of the participants worked as yoga teachers in addition to their other job or studies (Peter, Nicole, Leonie, and Mona); two interviewees were kite surfers working as instructors and technical product manager for kiting gear (Lina and Julia); Benjamin and Anne were both runners, one professionally, the other one as a (part-time) content creator and in her free-time; three participants were engaged in ball sports as athletes and coaches (Sarah, Adam, and Robin); and the remaining two interviewees worked as a man trailing instructor (man trailing is the search for a specific person with a dog; Karen) and as a climbing gym manager (Stephan). As diverse as their occupations and sports backgrounds may appear, the interviews revealed numerous similarities.

### Starting a Career as a Lifestyle Entrepreneur

The following part focuses on answering *RQ1:* What are the motivations to become a lifestyle entrepreneur and exchange the leisure activity for a professional career? When asked about their motivation to start an occupation within the sports industry, Mona stated, “I realized that yoga unites many of my interests” (yoga). Leonie confirmed this. She pointed out that yoga suited her to make a living and enjoyed helping other people with their lives and making them happy through the joint practice. Yoga allowed Leonie to work all over the world, which she appreciated a lot. Robin, a tennis coach, and lecturer echoed this, too: “Because it is fun and has always been a great passion of mine”.

These statements matched the idea that lifestyle entrepreneurs were not primarily driven by economic benefits but rather by personal values, interests, and passion (Andersson Cederholm, 2015; Guercini & Ceccarelli, 2020; Stebbins, 2009; Wallis et al., 2020). The dog rescue worker, Karen, was even motivated by her service to the community (Marcketti et al., 2006). The only exception seemed to be Benjamin, a professional trail runner, who started his career when his times had improved, giving him a better basis to negotiate with sponsors. He was at least partially motivated by economic factors. Additional factors, such as well-being or autonomy and freedom drove Benjamin.

Julia, a kitesurfer, noted that she returned to her job as a TV production manager once she had a baby. She stopped being a kite instructor and kite school manager because it did not provide her with the necessary job security: “The seasonal nature of the business, low salaries, and the physically demanding work do not offer much perspective in the long run. I did not have the opportunity to open a school myself back then. However, my biggest dream remains opening my wakeboarding or water sports center in the future”. Summing up, lifestyle entrepreneurs wanted to make a decent living (Deakins & Freel, 2006). In particular, lifestyle entrepreneurs’ work conditions and legal framework challenged this career path in the sports industry.

Another standard description for occupational devotees and lifestyle entrepreneurs was that the participants all turned their hobbies – in the case of this study, a sport – into their profession (Stebbins, 1992; Wallis et al., 2020). On average, they waited nine years before deciding to take this step, but they had a much longer background in their sports (averagely 19 years). The interviewees’ average age was 28 years when they started their sports careers (see Table 1 for a detailed overview). Robin started playing basketball at age six, and Karen picked up rescue dog work when she was 40 years old. The sporting career facilitated becoming a lifestyle entrepreneur: Robin’s entrepreneurial endeavors started when he was 18 years old, and Karen’s at age 47. Not all participants actively performed their sport as a core activity of their sports-related job: eleven participants work as instructors, coaches, players or run material tests on sports equipment. Of the remaining two interviewees, Stephan managed a climbing gym, and Anne maintained an Instagram channel about running – both missing the active element. Yet, they all actively and regularly engage in their sports, ranging from once a week with additional games on the weekends to daily practice.

Since the study participants all work in the sports industry, the emotional dimension plays a more critical role for these lifestyle entrepreneurs than it usually does in other sectors (González-Serrano et al., 2020; Guercini & Ceccarelli, 2020; Jones et al., 2017). For instance, Benjamin addressed this emotional dimension in the following statement: “It is my all-time favorite sport. (…) I’ve experienced moments, many moments, where I was running along a ridge all by myself with tears in my eyes”. A basketball coach stated that he still got goosebumps when he reminisced about past games his team had won (Adam, basketball). Stephan, the climbing gym manager, remembered, “once you finish a difficult route, a route on which you spent quite some time and effort, you will remember it. Those are the great moments in your life or whatever. I don’t know, but those are the things you won’t forget”.

### Well-being and the Sports Industry

**Fig. 1:**
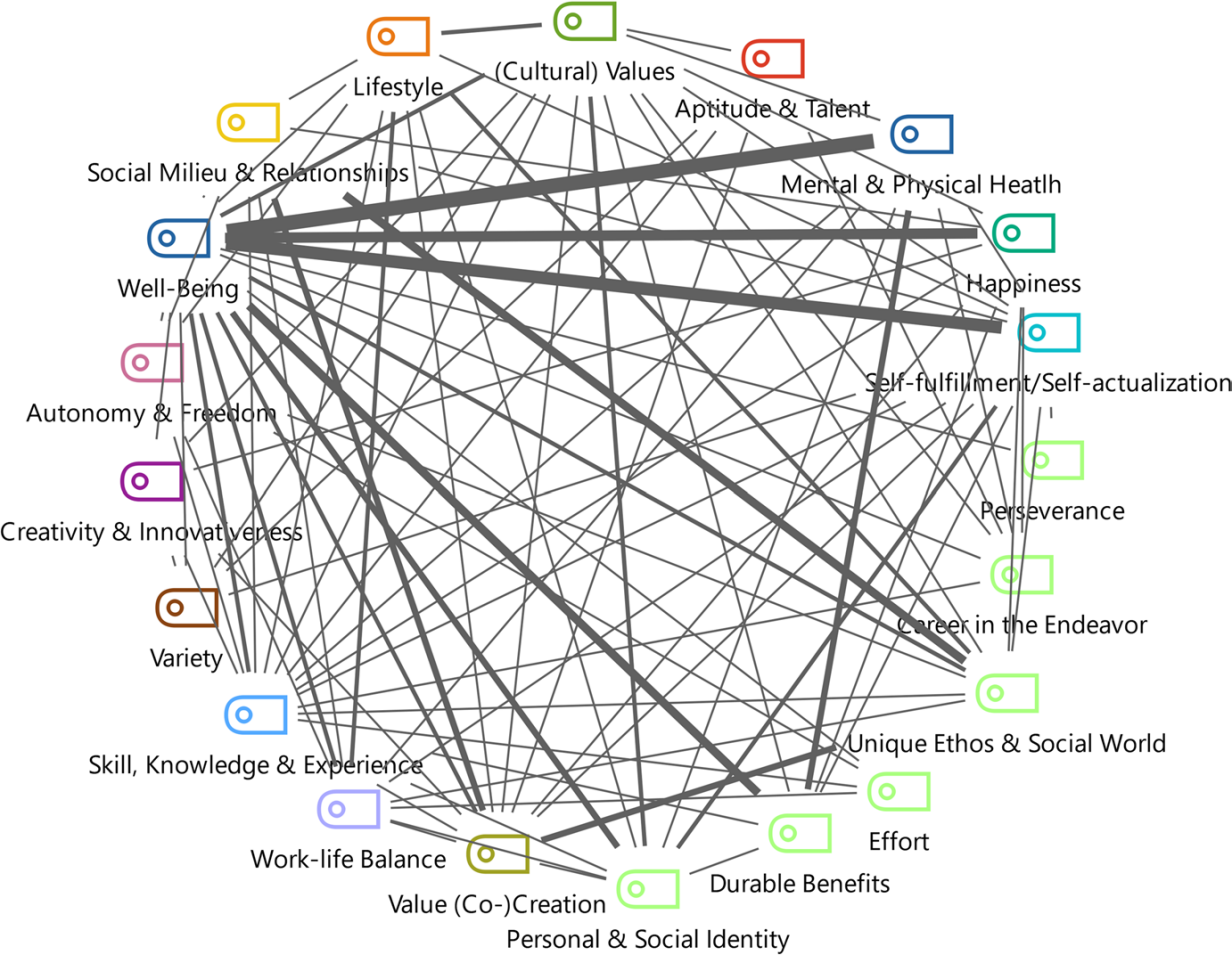
Overview of well-being and its sub-categories (i.e., self-fulfillment, mental & physical health and happiness) and their relationships with the dimensions of serious leisure, occupational devotion and co-creation

**Fig. 2:**
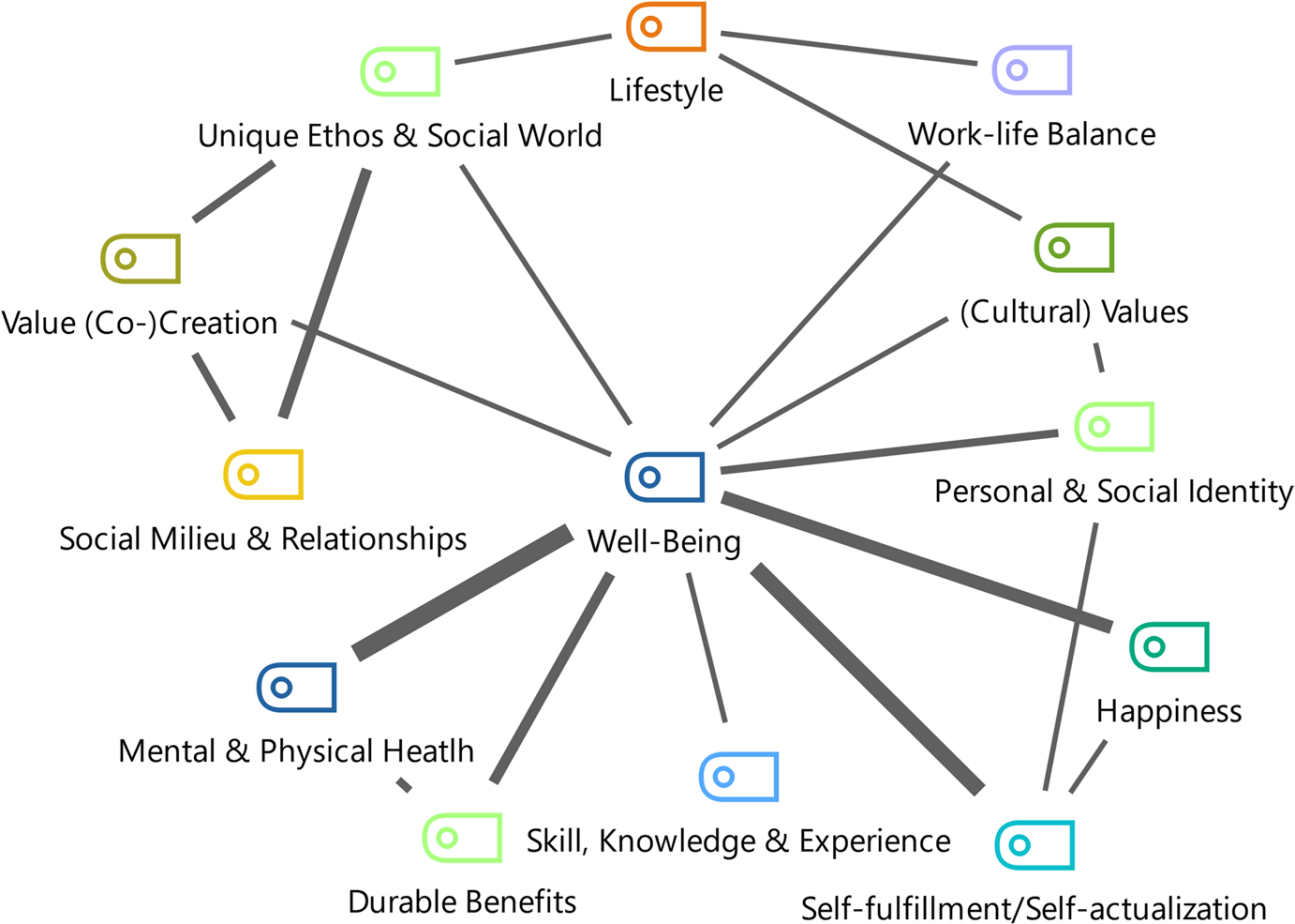
Core relationships of themes (at least six code overlaps in the interviews)

This section focuses on the participants’ well-being by examining *RQ2*: To what extent does the career as a lifestyle entrepreneur contribute to individual well-being? More and more people remodelled their work lives to improve their quality of life (Ateljevic & Doorne, 2000; Wang et al., 2019). Individual well-being played a significant role in this process. Well-being emerged as the most dominant theme, including happiness, self-fulfillment, and physical and mental health as sub-themes. These themes contributed to an individual’s quality of life, a core element of Stebbins’ occupational devotion (Stebbins, 2004b) and lifestyle entrepreneurship (Marcketti et al., 2006; Peters et al., 2009). Since the interviewees’ careers were in the sports industry, the themes might be displayed even stronger because of the field’s emotional dimension (González-Serrano et al., 2020; Guercini & Ceccarelli, 2020; Jones et al., 2017), as argued previously. Other core aspects influencing well-being were the durable benefits that participants drew out of their sports consumption in the form of serious leisure (Stebbins, 2007, 2009). Figure 1 represents the interviews’ code overlaps, meaning that individual statements could relate to several codes. The thickness of the bars refers to the frequency of these overlaps. For instance, happiness, mental and physical health, and self-fulfillment were subthemes of well-being. Figure 2 shows the most important relationships, mentioned six times or more.

Several interviewees highlighted happiness: “Ever since I practice yoga, I reached a different level of happiness” (Peter, yoga). Lina (kitesurfing) explained, “It’s a passion, which allows you to see special places, which makes you happy and you’re usually with friends. You simply have a great time.” Robin (tennis) stated, “I am always happy when I’m able to spend time on the tennis court”. The other interviewees also mentioned the term ‘happy’ several times and that their work elevated them. These statements stemmed from highly subjective experiences that individual people perceived as pleasant and interesting (Diener, 2000). Benjamin, the professional trail runner, described a five-hour trail run on a ridge with friends on a sunny day as pure joy from which he could draw energy for a few days, which kept him very happy. The same activity might be experienced quite contrary, maybe even negatively, by another person.

Some participants showed a solid connection to their sports-related job in the form of self-fulfillment. For ten of the eleven participants, self-fulfillment played a significant factor in starting a career in the sports industry as a lifestyle entrepreneur. Robin (tennis) and Mona (yoga) described it as the critical factor to pursue their lifestyle entrepreneurship. As part of the leisure industry, the sports industry represented a great channel for self-fulfillment (Neulinger, 1981). The strong effort and perseverance that the participants put into the sport amplified this. Accordingly, Sarah declared, “Football is something that defines my everyday life, my moods, my thoughts, my emotions, like nothing else in my life. At the moment, this is my highlight. All of these emotions you put into it, the effort you put into it. It’s more than an experience. It fulfills and defines me completely”. Other participants, for instance Julia (kiting), Lina (kiting), or Anne (running), compared their sport to “freedom” or a “feeling of being alive”. Adam, on the other hand, compared it to an intrinsic drive: “I got incredibly caught by basketball, and when you get addicted to a sport, you really want to get the best out of you. Then you feel like I have to go there, I want to get better, but I also want to pass it on as a coach. It’s intrinsic. I just have to do it”.

Similarly, Nicole stated that after having visited a yoga lesson and pushing herself beyond her limits, she felt surprisingly good without really knowing why. This experience ended up being a key reason for her later decision to start a career in yoga: “This is it, Nicole. This is what you want to do”. Peter (yoga) called it “an entirely new purpose in life”. These examples showed that self-fulfillment is a highly emotional factor, explaining why it contributed to a person’s quality of life (Huta & Waterman, 2014; Waterman, 2011). Figure 1 outlines that the relationship between well-being and (cultural) values was more robust than other relationships.

On the one hand, the results suggested the accruement of cultural capital required for sports entrepreneurship (Ratten & Tajeddini, 2019). On the other hand, this resulted from coding (cultural) values in the interviews according to Diener’s (2000) explanation: success, achievement, or individual personality. This definition was closely related to the factor of self-fulfillment, which is also perceived subjectively.

The third sub-theme of well-being in this analysis was mental and physical health. It predominantly appeared in the interviews with the four yoga instructors. Yoga was previously associated with mental health (Khalsa et al., 2012; Ross et al., 2013). Two interviewees had a medical background: Nicole worked as an alternative practitioner and had a university degree in medicine; Peter worked as a physiotherapist. Mona and Nicole claimed that they started their yoga careers for health reasons, such as back pain (Nicole) and high blood pressure (Mona). “To me, yoga is a tool for my body, a tool to find more peace of mind. So, to speak, using the body for personal development – mentally, emotionally, and physically. Also, to relax, to learn about oneself and others – both on the mat and in the teachings of yoga, the yamas and the niyamas,” explained Leonie and equally highlighted the health dimension. Peter elaborated on this by referring to yoga as “a new quality in his life – of physical and psychological nature” and that it had changed his life to the better. Ross et al. (2013) found that the more yoga was practiced, the stronger the belief yoga improves health. The effects of yoga on mental and physical health appeared during the participants’ practice and while they instructed a class: “Even when I teach, I seem to do yoga, even though I am not actively practicing. It is a form of activity that can stimulate the body, posture or body awareness, no matter if you actively do it or not” (Nicole).

However, physical and mental health did not only play a role for lifestyle entrepreneurs in yoga. It appeared in almost every interview, except for Robin (tennis), Karen (rescue dog work) and Lina (kiting). These health improvements were durable benefits of serious leisure. A systematic review confirmed that sports participation is beneficial for various health outcomes (Eime et al., 2013). Adam, who additionally worked as an elementary and high school teacher, mentioned that his job as a basketball coach offers him a “place to retreat [him]self and to regain energies for [his] everyday life”. The professional trail runner Benjamin made a surprising statement: “Ever since I started running professionally, interestingly enough, I no longer feel a need for a vacation. Because every week, or sometimes even daily, I experience my little vacations and adventures when I run up a new mountain. It feels like I’ve just been a thousand miles away. It has such a recreational and experiential factor for me”. Stephan even claimed still having a speech impediment if it had not been for climbing. Other interview statements referring to the mental and physical health theme included stress relief, self-confidence, learning how to deal with emotions, feeling comfortable and confident in their own body, mental strength, and general fitness.

To sum up, looking at Diener’s (2000) definition of well-being, the sports industry represented a work field with great potential to add to an individual’s well-being. The study participants associated positive emotions with their sports-related jobs and perceived it as attractive – not least because they were the ones to start a career as a lifestyle entrepreneur in the sports business to become more satisfied with their lives. The quality of life regarding work will always be higher if individuals can choose what they want to do and love what they do. Thus, factors such as creativity, autonomy, or lifestyle also contributed to an individual’s overall experience. These factors might contribute to the broader concept of well-being.

### Well-Being, Value Co-Creation, and Work-Life Balance

A good work-life balance was considered a primary driver for becoming a lifestyle entrepreneur. Thus, *RQ3* examined: What is the lifestyle entrepreneur’s work-life balance? Fig. 1 shows the links of value (co-)creation and work-life balance to well-being. It emphasized the essential role which value co-creation plays in sports entrepreneurship (Ratten & Jones, 2020). It was common for lifestyle entrepreneurs and occupational devotees to seek a better work-life balance through their chosen career path. Simultaneously, this often diminished the boundaries between leisure and work (Andersson Cederholm, 2015), summarized by Stephan as: “Climbing is my entire life. By now, I even do it for a living. […] But it’s always a balancing act between joy and business”. However, this was no contradiction for the interviewees because of their occupational devotion (Sun et al., 2020).

Stephan’s quote emphasized that his hobby became his business, as Wallis et al. (2020) outlined. Besides, it stressed the value of the activity and occupation simultaneously. Lifestyle preferences drove lifestyle entrepreneurs (Henderson, 2002). Lifestyle entrepreneurs considered their occupation as a lifestyle (Sun et al., 2020). The interviews confirmed this. Julia stated, “Living by the ocean and being able to get out on the water that much was what it so appealing” and Lina declared, “Once I had my teacher’s license, I went to Belize during winter to teach kiting and to enjoy the sun”. Lifestyle was named the most important factor for Benjamin regarding his career as a professional trail runner, as it was most important for him to spend much time in nature and the mountains.

Through these actions, lifestyle entrepreneurs simultaneously balanced their personal and professional lives (Andersson Cederholm, 2015; Ratten, 2018; Wallis et al., 2020). Yet, the lifestyle entrepreneurs mainly looked for their well-being and self-fulfillment, which they considered their ‘work-life balance’. Due to the sports industry’s emotional dimension, the lack of boundaries between the different life spheres might be more apparent than in other sectors (González-Serrano et al., 2020; Jones et al., 2017). Therefore, it was not uncommon for the interviewees to admit that their sports-related occupation defined their lives mostly or entirely. Sarah, the football player, described it as follows: “We practice every day, sometimes even twice a day, and on the weekends, we have games, sometimes all over Germany, so that we have to leave on Saturdays. So, every day is defined by football”. This highlighted that they understand their work-life balance differently than other people and that their occupation is truly their lifestyle (Sun et al., 2020).

Yet, they mostly perceived it as something positive compared to vacations, fun, recreation, excitement, or balance – matching Stebbins’ (2004a) definition of occupational devotion. Benjamin further described the overlaps of personal and professional spheres by mentioning that he was reasonably active on social media, where many people messaged him about his sport. However, at least one participant, Stephan, felt that his work was not truly balanced: “I didn’t climb for a long time when I opened this climbing gym. Just now I went climbing for 14 days like actually climbing (…) I don’t get away enough because of my job or don’t have the prospect of leaving for a week to find an interesting [climbing] project”.

The sports industry offered significant potential for value co-creation since it frequently involved multiple stakeholders, such as coaches, customers, or sponsors (Grönroos et al., 2015; Kelleher et al., 2019). This became evident throughout the interviews, as value co-creation was a common theme in the participants’ statements. Numerous times, it overlapped with the dominant theme of well-being, as presented in the following: “Although you are not experiencing the same practice as your students, you do hold the space, and when you sense that your students accomplish something, then I find a personal reward in it, too.” (Leonie, yoga) – an experience supported by all four yoga teachers. The two kite surfers, Lina and Julia, similarly explained that they experienced great joy when teaching their students or watching other kiters succeed.

However, the process of value co-creation was not limited to teacher-student environments but included experiences as a team or as part of a sports community. For instance, Robin (tennis) and Sarah (football) mentioned match experiences with their teams. Stephan (climbing) referred to the climbing community as “a big family that you meet everywhere, will likely see again and like because you share the same passion”, and Anne (running) spoke of an intense and unique experience of feeling united with thousands of people in a marathon. These descriptions highlighted the social dimension of value co-creation by lifestyle entrepreneurs in the sports business. Already within their consumption of sports as serious leisure, the sports’ social environment shaped the participants’ social world. Three interviewees (Leonie, Sarah, Anne) referred to social milieu and relationships as their primary drivers for their sporting careers. Overall, the interviews confirmed Ratten’s (2010) assumption that lifestyle entrepreneurs in the sports industry put a great emphasis on social capital.

### Implications

From a theoretical perspective, our findings confirmed the six features of occupational devotion: skill, knowledge or experience; variety; creativity or innovativeness; control; aptitude and taste; physical and social milieu (Stebbins, 2004a) for lifestyle entrepreneurs. The interviewees highlighted that skills, knowledge, and experience were highly relevant to being entrepreneurs in sport. Similarly, the lifestyle, relating to the physical and social milieu, was emphasized continuously. Thereby, the findings confirmed the linkage to the community (Ratten, 2018). Creativity and innovativeness were crucial to some interviewees, too. The other features, variety, control, and aptitude or taste, also played an important role, but to a lesser extent.

Emotions were a driver for lifestyle entrepreneurs, as our results suggested. This confirmed previous findings (Sun et al., 2020) and equally highlighted the importance of well-being. The results also revealed a connection between occupational devotion and well-being, which complemented the theory. The relationship between occupational devotion and well-being was strongest for those involved in yoga. This can be related to the nature of yoga. The relationship between occupational devotion and well-being was expected, given that lifestyle entrepreneurs strive for a good work-life balance through constant negotiations. Our study confirmed this for various sports. However, the perceptions of work-life balance were different for lifestyle entrepreneurs. It became obvious that their profession was synonymous with their lifestyle (Sun et al., 2020) and helped them strive for self-fulfillment and well-being. Most respondents negotiated their time allocation to work and their personal life with the family to be happy with it. Yet, it is unknown how they negotiated their time allocation regarding space (e.g., when they decide to be in their studio, on the rock, in the gym, and the distance from where they live).

From a practical perspective, lifestyle entrepreneurs should experience more acknowledgement and support. They co-created value with members of the community and thereby developed social capital. Thus, support for lifestyle entrepreneurs is recommended as they provided community benefits. Lifestyle entrepreneurs were innovative and constantly adapting to change (Ratten, 2020a). Other sports organizations can learn from them how to react to crises, as suggested by Ratten (2020a). Lifestyle entrepreneurs strived for happiness and well-being through their businesses.

Even though being a lifestyle entrepreneur led to better well-being by combining their leisure passion with an occupation, other life areas (e.g., family responsibilities, household, friends) might be harder to balance. The local chambers of commerce should offer special workshops for lifestyle entrepreneurs to gain more profound knowledge regarding business practices and balancing work and leisure when they overlap. The majority of lifestyle entrepreneurs interviewed were not aware of their workload and the overlaps, which could wear them out. Therefore, preventive measures in this regard are recommended helping lifestyle entrepreneurs to achieve well-being more easily.

Policymakers should also commend the lifestyle entrepreneurs and their engagement as they contributed to an active and sportive community. Yet, the careers of lifestyle entrepreneurs depended highly on the demand of customers. By pursuing their profession, they delivered sports services for various target groups. Consequently, they supported sports participation and social interactions, which positively influences the participants’ health. Sports and physical activity had physiological and psychological health benefits and prevented common health problems (Malm et al., 2019). Therefore, lifestyle entrepreneurs were beneficial for the community as they decisively contribute to sports within the community. Appreciation of the community would be good recognition for the lifestyle entrepreneurs.

### Limitations and Future Research

The study’s findings offered a better understanding of why lifestyle entrepreneurs pursue a professional career in their sports, full or part-time. The relatively small number of interviews proved very useful in analyzing the qualitative interviews in-depth and with high precision, yet it also posed a limitation to this study. We can only claim generalizations on the studied topic to some extent, which is why we recommend a greater sample size for future studies in this field. However, this study aimed not to generalize but to study the motivation of sport-based lifestyle entrepreneurs and explore the effects on the individuals’ perception of well-being. Further, the interviews revealed many personal aspects and shared values to which many – we believe – will be able to relate to and transfer to their own lives.

The authors understood the lifestyle entrepreneur as self-employed entrepreneurs. Yet, lifestyle entrepreneurs do not need to own and operate their company. Since national legislation differs from one country to another, this generalization applied throughout the paper. For instance, in Germany, one does not need to own a company to be self-employed. Considering geographical breadth, we acknowledge that the data were collected in Germany. Therefore, the results will likely be relevant to other Western countries – despite special features from Germany – but not to all countries across the world.

Due to the complexity of the concept of lifestyle entrepreneurship (Wallis et al., 2020), we highly recommend future research in this area. Further studies should use larger sample sizes to generalize results and compare self-employed and employed sports lifestyle entrepreneurs, their motives, and their state of well-being. Future research could compare different types of sports, such as team sports vs. individual sports, or health sports (e.g., yoga) vs. competitive sports (e.g., triathlon), or nature-based (e.g., surf, hike, climb) vs. traditional (e.g., tennis, athletics, swimming) sports. Other geographic contexts to what extent the environment as space (or experiencescape) in which the occupational devotion occurs is meaningful for lifestyle entrepreneurs should be investigated. It would also be interesting to shed more light on the negotiations of lifestyle entrepreneurs’ time allocation to their occupation and other areas in their lives (family, sleep, other leisure activities – if any, etc.).
